# Editorial: Machine learning advancements in pharmacology: transforming drug discovery and healthcare

**DOI:** 10.3389/fphar.2025.1583486

**Published:** 2025-03-07

**Authors:** Moom Rahman Roosan, Ramgopal Mettu

**Affiliations:** ^1^ Pharmacy Practice Department, Chapman University School of Pharmacy, Irvine, CA, United States; ^2^ Department of Computer Science, Tulane University, New Orleans, LA, United States

**Keywords:** machine learning (ML), artificial intelligence in healthcare, computational drug design and discovery, drug repurposing 4, predictive analytics in healthcare, survival prediction models

## Introduction

In recent years, the integration of machine learning (ML) into pharmacology has revolutionized how we approach drug discovery, disease modeling, and therapeutic development. By leveraging vast datasets and computational power, ML has enabled researchers to uncover patterns, predict outcomes, and accelerate drug development processes that were previously unimaginable. This Research Topic on “Machine Learning Advancements in Pharmacology” features five impactful studies that highlight the diverse applications and potential of ML in this field. These contributions, encompassing original research and a systematic review, exemplify the transformative role of ML in addressing some of the most pressing challenges in pharmacology.

## Drug discovery

Yao et al. conducted a bibliometric analysis of graph neural networks (GNNs) in drug discovery between 2017 and 2023. Their findings reveal significant contributions from China and the United States in areas such as drug-target interaction prediction, drug repurposing, and drug-drug interaction analysis. While GNNs demonstrate promising applications, challenges such as data availability, ethical considerations, computational demands, and the need for interpretability remain barriers to widespread adoption.

Kapanaiah et al. introduced an interpretable ML approach to analyze multi-site electrophysiological data, revealing the neural effects of dopaminergic compounds. Authors administered dopaminergic antagonists—clozapine, raclopride, SCH23390—and the agonist amphetamine to mice, recording local field potentials across cortico-thalamo-hippocampal networks. The ML model identified distinct alterations in neural activity and connectivity patterns specific to each compound, providing insights into their circuit-level mechanisms. This methodology offers a robust framework for characterizing the neural signatures of neuropsychopharmacological agents, potentially enhancing our understanding of their therapeutic and side effect profiles.

Kim et al. presented a novel structure-based inference method that predicts protein-ligand binding affinity by utilizing multiple molecular docking poses for each complex. Their approach integrated multi-instance learning (MIL) with an attention network, allowing for accurate predictions without relying on co-complex crystal structures, which are often unavailable.

MIL, a weakly supervised learning paradigm, is particularly effective when only aggregate labels are available rather than labels for individual data points. The authors capitalized on MIL’s ability to process multiple docking poses to improve binding affinity predictions, even in the absence of experimental structural data. This significantly enhanced virtual screening processes by accounting for structural flexibility and uncertainty.

The model was validated using PDBbind and datasets containing compounds targeting the main protease of SARS-CoV-2, demonstrating competitive performance compared to models that require crystal structures. By leveraging docking poses, this method expanded the applicability of binding affinity predictions to previously inaccessible protein targets, marking a major advancement in AI-driven drug discovery and virtual high-throughput screening.

## Survival and drug efficacy prediction

Li et al. introduced a novel Triglyceride-Inflammation (TI) Score to predict overall survival (OS) in patients with nasopharyngeal carcinoma (NPC) in their retrospective study. By analyzing pre-treatment levels of triglycerides and inflammatory markers—namely neutrophil-to-lymphocyte ratio (NLR), lymphocyte-to-monocyte ratio (LMR), and platelet-to-lymphocyte ratio (PLR)—the researchers employed a random survival forest (RSF) algorithm to develop the TI Score. The model demonstrated strong predictive performance, with concordance indices (C-index) of 0.806 in the training set and 0.759 in the validation set. Time-dependent receiver operating characteristic (ROC) curves further supported its accuracy, with an area under the curve (AUC) values at 1, 3, and 5 years indicating excellent prognostic capability. Notably, higher TI Scores correlated with advanced disease stages (T3-T4 or M1), elevated NLR and PLR, and reduced LMR, underscoring the interplay between lipid metabolism and systemic inflammation in NPC progression. The study also found significant interactions between triglycerides and NLR, suggesting that triglyceride metabolism may influence immune responses in NPC. These findings highlight the potential of the TI Score as a convenient and cost-effective prognostic tool, offering valuable insights for personalized treatment strategies in NPC patients.

Zhang et al. developed an ML algorithm designed to predict the efficacy of chidamide in patients with angioimmunoblastic T-cell lymphoma (AITL). Researchers analyzed data from 183 newly diagnosed AITL patients across three centers in China, identifying key prognostic features influencing treatment outcomes. The ML model demonstrated high accuracy in forecasting patient responses to chidamide, offering a potential tool for personalized treatment strategies in AITL. This approach underscores the promise of integrating ML into oncology to enhance therapeutic decision-making and improve patient prognosis.

## Themes and emerging trends

Across these studies, a few key themes and trends emerged for ML in pharmacology and healthcare. First, all of the contributions in this issue demonstrate that the integration of ML with pharmacology has bridged disciplines, combining computational expertise with domain-specific knowledge in pharmacology. ML-driven advancements in rational drug design and high-throughput screening are becoming increasingly mainstream and accelerating pace of discovery. Second, from survival prediction to drug efficacy, ML has demonstrated its potential to tailor interventions to individual patients, advancing the concept of precision medicine (e.g., Li et al. and Zhang et al.). Third, several contributions highlight the importance of interpretability in ML models, ensuring their applicability in clinical and regulatory contexts (e.g., Li et al. and Kapanaiah et al.). These trends illustrate the emerging role of ML in pharmacology and underscore the need for continued innovation in algorithms, data quality, and model validation. As ML gains traction in healthcare, maintaining model interpretability, interoperability and data quality will be crucial for its widespread adoption.

## Challenges and future directions

While the studies featured in this issue demonstrate significant progress, challenges remain. High-quality, annotated datasets are critical for training robust ML models. Addressing gaps in data Research Topic and standardization is essential. The ML models must perform well across diverse populations to ensure model generalizability for real-world applications. For ML-driven insights to transition into clinical practice, their interpretability and validation must meet regulatory standards. As ML becomes more integrated into clinical decision-making, addressing bias issues and ensuring models’ ethical use are paramount. Strategies for bias detection and mitigation should be prioritized to prevent disparities in drug development, patient treatment, and healthcare outcomes. Future research should aim to address these challenges while continuing to explore innovative applications of ML in pharmacology.

## Conclusion

The articles featured in this Research Topic illustrate the significant potential of machine learning in pharmacology. From accelerating drug discovery with advanced computational models to personalizing cancer treatment and unraveling the neural effects of pharmacological compounds, these contributions showcase the expanding role of ML in revolutionizing the field. As we continue to innovate, the collaboration between computational scientists and pharmacologists will be key to unlocking new therapeutic possibilities. We hope this issue inspires further research and collaboration at the intersection of ML and pharmacology.

